# Hesperidin from Orange Peel as a Promising Skincare Bioactive: An Overview

**DOI:** 10.3390/ijms25031890

**Published:** 2024-02-04

**Authors:** Cristina V. Rodrigues, Manuela Pintado

**Affiliations:** CBQF—Centro de Biotecnologia e Química Fina—Laboratório Associado, Escola Superior de Biotecnologia, Universidade Católica Portuguesa, Rua Diogo Botelho 1327, 4169-005 Porto, Portugal; civrodrigues@ucp.pt

**Keywords:** hesperidin, orange peels, food industry byproducts, renewable bioactive compounds, sustainable skincare formulations

## Abstract

The pursuit for better skin health, driven by collective and individual perceptions, has led to the demand for sustainable skincare products. Environmental factors and lifestyle choices can accelerate skin aging, causing issues like inflammation, wrinkles, elasticity loss, hyperpigmentation, and dryness. The skincare industry is innovating to meet consumers’ requests for cleaner and natural options. Simultaneously, environmental issues concerning waste generation have been leading to sustainable strategies based on the circular economy. A noteworthy solution consists of citrus by-product valorization, as such by-products can be used as a source of bioactive molecules. Citrus processing, particularly, generates substantial waste amounts (around 50% of the whole fruit), causing unprecedented environmental burdens. Hesperidin, a flavonoid abundant in orange peels, is considered to hold immense potential for clean skin health product applications due to its antioxidant, anti-inflammatory, and anticarcinogenic properties. This review explores hesperidin extraction and purification methodologies as well as key skincare application areas: (i) antiaging and skin barrier enhancement, (ii) UV radiation-induced damage, (iii) hyperpigmentation and depigmentation conditions, (iv) wound healing, and (v) skin cancer and other cutaneous diseases. This work’s novelty lies in the comprehensive coverage of hesperidin’s promising skincare applications while also demonstrating its potential as a sustainable ingredient from a circular economy approach.

## 1. Introduction

Peer and self-perception are concepts that have been playing a major role in our daily life, as a society, throughout time [1]. However, in the last few years, most people have been searching for ways to improve their skin and overall appearance in a fast and efficient way due to an increasingly stressful lifestyle while embracing a healthy skin complexion [2]. Skin is the largest organ in the human body, being subjected to various external factors and pathogens that can compromise its well-being. Since factors such as sun damage, originating from ultraviolet (UV) radiation; environment pollution; bacteria and fungus; or common daily habits (e.g., poor diet, alcohol ingestion, and smoking) might impair the highly complex cutaneous function, various skincare products (e.g., cosmetics and pharmaceuticals) can be used to minimize or even reverse their effects [3,4]. In this way, some of these deleterious factors might accelerate the skin’s aging process, which can cause various alterations, such as protein (e.g., elastin and collagen) deterioration by metalloproteinases (MMPs), and the activation of pro-inflammatory cytokines, among other molecular responses, ultimately culminating in the disorganization of the extracellular matrix (ECM); this disorganization can generate skin inflammation, wrinkles, skin elasticity loss, hyperpigmentation, and dehydration, among other issues [3,4,5,6]. On the other hand, skincare products can be divided into distinct categories according to their composition, pathway of action, and effects, namely cosmetics, cosmeceuticals, nutricosmetics/nutraceuticals, and skincare pharmaceuticals/therapeutic agents [7,8]. Briefly, cosmetics focus mainly on aesthetics, and they do not have the capacity to alter the skin’s structure or function but rather provide a temporary superficial enhancement (e.g., moisturizers). Regarding cosmeceuticals, although their definition has been a topic of discussion over the years, they can be considered to be a hybrid between cosmetics and pharmaceuticals, intended to have both a cosmetic and, to some extent, a therapeutic effect on the skin, leading to skincare benefits such as a reduction in the appearance of wrinkles [7,8]. Also, skin-related nutricosmetics or nutraceuticals involve the use, for instance, of oral supplements to promote skin health, being composed of molecules that are believed to support skin health when ingested [7]. However, skincare pharmaceuticals/therapeutic agents or dermatological drugs are formulated as therapeutics, using active pharmaceutical molecules, to address complex skin conditions, such as psoriasis, eczema, or chronic wounds, and they are highly regulated [8].

Overall, skincare goods can target many of the above-mentioned problems, which can be economically translated into a high-value market. As an example, according to Statista [9], the global cosmetics market has been consistently growing since 2004, and it is expected to generate a total sum of nearly USD 129 billion in revenue by 2028. In addition, as reported by Market Research [10], the dermatological therapeutics market is also expected to reach around USD 53.5 billion in revenue by 2027. Due to the increasing interest regarding this matter, a demand for new active ingredients and resources that hold the potential to be incorporated into skincare products and aim for those concerns has risen [4].

Consumers have been progressively reaching out for products based on compounds extracted from natural sources (e.g., plant extracts), favoring “clean” skincare alternatives [1,11]. Taking into account these trends and consumer needs, the skincare industry has also searched for new natural molecules with outstanding properties in an attempt to innovate and come up with novel solutions in this field [12]. Adding to this collective interest from both consumers and the industry, it is important to note that diverse environmental issues regarding consumption and waste are challenges that represent an increasing burden on the environment, and these challenges need to be addressed [1,13]. One example of a viable solution for this matter is the application of the circular economy approach, comprising the incorporation into skincare formulations of active molecules extracted from agro-food by-products with potential properties that might benefit the skin. In this way, biomass that would be otherwise wasted, leading eventually to environmental problems, can be reused to obtain a value-added product [3,14].

All over the world, the processing of citrus by the food industry is expected to be up to 120 million tons every year [14]. Among citrus fruits, oranges, in particular, are one of the most produced and consumed fruits worldwide [15,16,17]. Therefore, large amounts of by-products (e.g., seeds, peels, and pulp) result from this sector [18,19,20]. It is estimated that as much as 45% to 50% of the fruit is discarded [3]; however, these by-products are extremely abundant in phenolic compounds, which can be extracted, purified, and further applied in skincare formulations [16,17,21]. Phenolic compounds, such as flavonoids, phenolic acids, lignans, tannins, and stilbenes, are compounds that possess one or more hydroxyl substituents [22,23]. Flavonoids are one of the most researched groups of polyphenols; however, more studies regarding their absorption and effects on the skin are needed [22,23,24]. A flavonoid highly present in diverse citrus fruits—particularly in orange peels—is hesperidin [4,14,25,26]. Hesperidin was first discovered in 1872 by the French chemist Lebreton and was initially named “vitamin P” [22]. This flavonoid is structurally defined by an aglycone known as “hesperetin”, which binds to it a 7-positioned 6-O-α-L-rhamnosyl-D-glucose moiety via a glycosidic linkage [22,23,24]. Hesperidin is considered a compound of extreme interest, since it presents a wide range of potentially valuable properties to the skin, including antioxidant, photoprotective, anti-inflammatory, anticarcinogenic, and antibacterial activities [14,26,27]. Moreover, hesperidin concentration has been found to be higher in orange peels than in juice or seeds [28]. Therefore, the extraction of hesperidin from orange by-products, specifically peels obtained from agro-food wastes, can result in the formulation of a high-value product for the skincare industry [17,29] while addressing environmental challenges [14,26].

To the best of our knowledge, there are no reviews that have provided such an extensive overview of the research evolution on hesperidin valorization, extraction, and biological properties, specifically for its application in skincare, within the last 10 years. Therefore, this review aims to outline and offer a new perspective on the research progress regarding hesperidin from citrus agro-food wastes within the last decade and emphasizes particularly the extraction methodologies and biological properties of hesperidin from orange peels as one of the most abundant citrus by-products. Beyond this, the present review explores, in a comprehensive way, the potential of hesperidin as a sustainable bioactive ingredient for vast skincare applications. Hence, the novelty of this work lies not only in the exhaustive coverage of hesperidin’s diverse applications in the skincare industry but also in showing the opportunity that the usage of hesperidin represents to transform discarded orange peels into a valuable resource for promoting skin health and well-being. Thus, in an era seeking sustainable solutions, this review highlights environmentally conscious approaches to skincare research based on a circular economy approach.

## 2. Studies Published in The Last Decade—An Overview

### 2.1. Research Methodology

In this review, papers on hesperidin from orange peels published in the last decade were obtained through the Web of Science platform, using the keywords “hesperidin”, “orange”, “extraction”, “skin”, “aging”, “cosmetic”, “wound healing” and “skin cancer” while applying the filters for “all databases” and “topic”. This approach ensured the inclusion of all articles, review articles, abstracts, clinical trials, and patents available in the databases containing these keywords.

The exclusion criteria for the search were non-English written documents and records that did not analyze the extraction methods from orange peels or study the bioactivities of hesperidin on the skin.

### 2.2. Results

**Figure 1:**
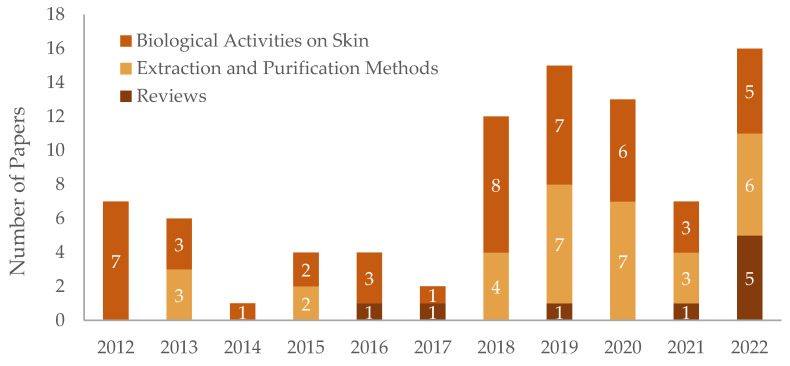
Number of papers, from the last decade, related to hesperidin extraction and purification methods from orange by-products, namely peels, and the application of its functionality to the skin. The papers were analyzed and selected accordingly with the specified criteria. The considered articles were divided into the themes “Reviews”, “Extraction and Purification Methods” and “Biological Activities on Skin”.

**Figure 2:**
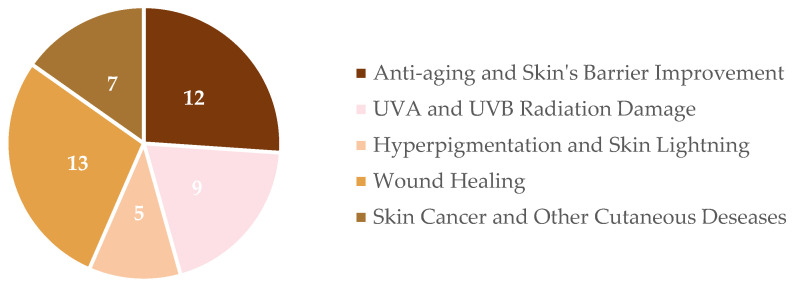
Number of papers related to hesperidin’s biological activities on skin from the last decade. The works that followed under the scope of “Biological Activities on Skin” (previously defined) were subdivided into “Anti-Aging and Skin’s Barrier Improvement”, “UVA and UVB Radiation Damage”, “Hyperpigmentation and Skin Lightning”, “Wound Healing” and “Skin Cancer and Other Cutaneous Diseases”.

In total, 256 papers resulted from the research conducted by following the parameters described above. However, only 87 papers were carefully selected, taking into consideration the exclusion criteria (Figure 1). The selected papers were separated into three main categories: “Reviews”, “Extraction and Purification Methods” and “Biological Activities on Skin”. Moreover, the last category was subdivided into different topics to further analyze these subjects, namely “Anti-Aging and Skin’s Barrier Improvement”, “UVA and UVB Radiation Damage”, “Hyperpigmentation and Skin Lightning”, “Wound Healing” and “Skin Cancer and Other Cutaneous Diseases” (Figure 2).

## 3. Hesperidin Extraction and Purification from Oranges’ Peel Waste

**Figure 3:**
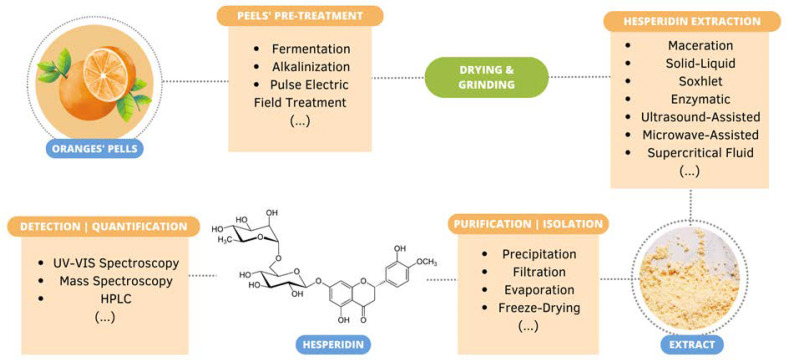
Schematic representation of hesperidin’s extraction and purification process from orange peels [18,28,32,34,35,36,37,38,39,46,47,48,49,50,52,54,57,58,59,63,65,67,68,69,70,71].

Diverse extraction and purification methods have been described for obtaining hesperidin extracts (Figure 3). For any type of compound intended to be applied in the healthcare and wellbeing industry, namely in the skincare field, its safety and non-toxic nature need to be guaranteed [30]. Therefore, well-thought-out optimal methods need to be chosen, depending on the final extract application [28].

To improve the yield of phenolic compounds, some supplementary steps can be added before extraction (pre-treatment). A common procedure is the drying of the orange peels [3,31], which allows a faster extraction process [32]. Another described approach is fermentation, aiming to increase the quantity of orange peel secondary metabolites, including polyphenols such as hesperidin, and decrease sugar content [33,34]. This methodology has been reported by Kyung and colleagues [35], who applied sugars, lactic acid bacteria, or even mushroom mycelium to ferment *Rutaceae* fruit extracts. Complementary extraction methods also tested include alkaline treatment, which removes the pectin within the extract by making it insoluble [28], facilitating extract purification later on; the application of a pulsed electric field for overall improvement in hesperidin’s yield and enhancement of biological properties (e.g., antioxidant capacity) [36]; and the instant controlled drop method, allowing easier accessibility to biomolecules in the proper extraction step [37]. Following pre-treatment completion, it is common to subject the samples to grinding and homogenization, which are often achieved through freeze-drying [38,39,40,41].

Regarding the extraction process itself, the recovery of flavonoids from agro-food by-products is usually accomplished through solid–liquid extraction. This process is typically carried out by using organic compounds, such as ethanol and methanol solutions, to obtain the extracts [18,42]. The obtention of flavonoids, namely hesperidin, through water extraction has also been reported by some studies [43]. However, considering the poor solubility of hesperidin in water (yield around 7.06%) [44], ethanolic extracts are claimed to yield higher quantities (around 40%) [28,45]. Nonetheless, processes based on green chemistry have been emerging. This technology is considered valuable since it allows the reduction in energy and additives usage, holds less complex operating conditions, and is easier to scale up [18]. In this way, methods such as pressurized liquid extraction [46,47], water bath and ultrasound-assisted extractions [48,49,50,51], hydrodynamic cavitation [52], steam explosion followed by water extraction [53] and subcritical water extraction [38,54], among others (Figure 3), do not require the application of volatile organic solvents. Other commonly used methods, which are being replaced by more advanced and efficient procedures, include maceration [55], Soxhlet-mediated extraction [56,57], and enzymatic extraction. The latter was tested by Madeira and colleagues [58], who specifically applied cellulase, pectinase, and tannase to carry out the process. In addition, alternatives like solid-phase extraction can also be applied to obtain hesperidin-rich extracts [38].

Moreover, some aspects that can be targeted for optimization, as they are reported to have a high impact on the final hesperidin yield, include the solvent type, the sample-to-solvent ratio [38,59], time, pH, and temperature conditions of the process [60,61].

Considering the isolation and purification of the extracts, several methods can be applied to obtain the aimed compound, hesperidin. For this purpose, centrifugation [62], membrane filtration technology [18], acid precipitation [63,64], and high-performance liquid chromatography (HPLC) [28] are examples of methods that can be used for the isolation and purification of hesperidin. Zhang and colleagues [65] used an evaporation technique to concentrate the obtained extracts’ pellets after centrifugation; then, they resuspended them to apply different solvents, obtaining three distinct fractions. This type of procedure might be useful to further improve the purity of the final product.

After the isolation and purification steps, techniques such as evaporation and freeze-drying, for instance, can be used to concentrate and preserve hesperidin [39,44,66]. Additionally, the main techniques usually applied for its detection and relative quantification are HPLC, UV-VIS, Fourier transform infrared spectroscopy (FT-IR), mass spectroscopy, and 1H nuclear magnetic resonance spectroscopy (1H-NMR) analysis [28,38,65].

## 4. Application of Hesperidin’s Biological Activities on Skincare

Hesperidin has been reported to present various biological activities with the potential to be applied in the skincare field, either in a cosmetic, cosmeceutical [72], or pharmaceutical/therapeutical context [73], as it shows a considerable number of characteristics that can work toward skin improvement [38,74]. The chemical composition of the flavonoids might vary on hydroxylation and polymerization degree, substitutions, and conjugations [38]. Additionally, the molecular weight of hesperidin is 610.57 g/mol, with its melting temperature being around 255 °C. It is also claimed that hesperidin can maintain its stability for up to two years when stored at −20 °C [4]. Hence, hesperidin holds a unique structure that allows this compound to have several cutaneous pathways of action, enabling it to target multiple skin issues [38].

Despite all the potential bioactivities of hesperidin for skincare, its formulation continues to represent a challenge due to its solubility constraints. The main problems include the poor solubility of hesperidin in water and its low lipophilic character, which leads to reduced skin absorption [22]. However, new technologies have been developed aiming to overcome this issue.

Therefore, this section has the objective to review, in detail, the bioactivities reported from hesperidin, specifically the one from oranges’ peels, and its proposed potential application within the skincare field. This includes the levels of hesperidin, assays, testing models, bioavailability assessments, and formulation techniques that have been used along with some molecular mechanisms that can be regulated by this flavone on the skin.

### 4.1. Antiaging and Skin’s Barrier Improvement

Intrinsic skin aging is a natural process influenced by numerous endogenous and exogenous factors. Although inevitable, the process of skin aging can lead to a certain level of discomfort due to skin dehydration and roughness. Moreover, concerning aesthetics, signs of skin aging might have a negative impact on people’s self-image [75,76]. In this way, the development and usage of skincare products containing compounds capable of targeting those inherent aging signs, such as the formation of wrinkles, laxity, dryness, or atypical pigmentation alterations, might be helpful [77].

Considering intrinsic factors, the concepts of aging and oxidative stress have always been interconnected [3]. The aging of cutaneous tissue involves the degradation of its ECM by MMPs, among other enzymes (e.g., elastase) [3,78,79]. MMPs normally contribute to the natural remodulation of ECM; however, when overexpressed, they promote skin damage and accelerate the skin aging process. ECM degradation includes several alterations, such as loss of skin proteins (mainly collagen and elastin) and reactive oxygen species (ROS) production, leading to DNA and mitochondrial damage, as well as lipid peroxidation [3,6,75,78,80]. ROS can also alter skin structure by breaking cell adhesion and the basement membrane structure. All these alterations can compromise the skin barrier, leading to moisture loss [79]. Logically, compounds that can target any of these molecular mechanisms have the potential to delay skin aging either through the regulation of various intracellular pathways or by direct improvement of the skin’s barrier. For instance, it was described that hesperidin can target MMPs by instigating the expression of metalloproteinase tissue inhibitors (TIMPs). Furthermore, hesperidin can present a protective activity against ROS through direct interaction by inducing the activation of antioxidant enzymes or even by oxidase inhibition [63].

As mentioned before, various external factors can also be involved in the aging process of human skin, one of them being the environment. Highly polluted environments subject the skin barrier to stressing conditions [81]. Fernando and colleagues [81] described the effects of particulate matter, a group of liquid and solid atmospheric compounds, on the skin. According to their findings, these compounds damage the skin by inducing senescence and inflammation by producing ROS and triggering apoptosis [81].

Since hesperidin is a flavonoid, it is claimed to possess antioxidant activity [56]. In this way, hesperidin acts as a scavenger against ROS, such as 2,2-diphenyl-1-picryl-hydrazyl-hydrate (DPPH) and 2,2′-azinobis-(3-ethylbenzothiazoline-6-sulfonic acid (ABTS) free radicals [35,82]. This activity is related to the releasing of protons from this flavone, modulating the radicals’ synthesis toward the hesperidin structure [27,38]. Furthermore, it was suggested by Baker and colleagues [27] that this is regulated through the ERK/Nrf 2 signaling pathway. Other examples of activities of hesperidin from orange peels that can counteract skin aging and that can be incorporated into cosmetic or cosmeceutical formulations are typified in Table 1.

Regarding hesperidin formulations within an antiaging context, it was reported that its combination with topical clobetasol can be beneficial to restore skin barrier functions [83], which may also be related to the Nrf2 pathway [84,85].

Skin photoaging promoted by ultraviolet (UV) radiation is also a relevant and highly researched topic related to skin aging [12]. Therefore, the effects of hesperidin on overcoming the consequences of skin exposure to UV radiation are described in depth further ahead.

**Table 1:** Biological activities of commercial and orange peel’s hesperidin related to skin anti-aging and skin’s barrier improvement.

Hesperidin/ Extract Type	Hesperidin Levels Tested	Testing Model	Main Biological Properties	Formulation	Potential Applications	Molecular Pathways/ Other Relevant Properties	Ref.
Commercial hesperidin	≈35–70% (*w*/*v*)	In vitro non-cellular assays	Radical scavenging activity increase in a dose-dependent manner.	Complex of hesperidin with modified silica (1:1, 1:2 or 2:1 (*w*/*w*))	Cosmetics/ Cosmeceuticals	Complex’s antioxidant properties were higher than free hesperidin	[23]
*Rutaceae* fruits extract	1254.67 μg/mL	*S. epidermidis* *E.coli* *P. acnes*	Radical scavenging activity; Antibacterial activity against *S. epidermidis*, *E. coli* and *P. acnes.*	Mixed *Rutaceae* fruits hesperidin-rich ethanolic extract fermented with mushroom mycelium	Cosmetics/ Cosmeceuticals	N/A	[35]
*C. unshiu* peel extract	0.25–1% (*w*/*v*)	HDFs cells	MMP-1 expression decreases in a dose-dependent manner; Senescent cells decrease; Collagen biosynthesis increases.	*C. unshiu* peel extract fermented with *S.commune*	Cosmetics/ Cosmeceuticals	N/A	[44]
*Citrus sinensis* peel bagasse	Corrosion tests: 100 µg/mL Collagenase assay: 0.08–0.9 mmol/L	FTS cells	Chelation activity; Collagenase inhibition; Antioxidant activity.	Nanoemulsion: hesperidin; glycerol; orange oil, poloxamer (Pluronic F127); and water. Nanoemulsion Silky Cream: BisPEG/PPG-16/16 PEG/PPG-16/16 dimethicone; caprylic/capric triglyceride (1%); muru muru butter (1%); cupuaçu butter (1%); andiroba oil (3%); GMS (2%); cetostearyl alcohol (3%); mineral oil (5%); carbopol ULTREZ 10 (0.2%); nano-emulsion (79.8%); triethanolamine (5 drops); vit E-acetate (0.05%); ethylhexylglicerin, phenoxy-ethanol (0.05%)	Cosmetics/ Cosmeceuticals	Modulation of MMPs activities	[63]
Commercial hesperidin	50 µM	HaCaT cells exposed to PM2.5	Intracellular ROS levels reduction; Apoptotic index reduction; Protein carbonylation and intracellular vacuoles accumulation reversion; Reversion of elevated mitochondrial depolarization; Overall inhibitory effect on PM2.5-induced skin senescence and aging.	N/A	Cosmetics/ Cosmeceuticals	Cell viability restoration via PI3K/Akt activation; MAPK activation and autophagy/apoptosis-related protein expression mitigation; Increase in the anti-apoptotic protein Bcl-2 expression; Cell arrest in the G0/G1 phase decrease; β-galactosidase activity and MMP-associated senescence decrease; Oxidation effects decreased by c-Jun and c-Fos protein levels reduction	[80,81]
Commercial hesperidin	0.02% (*w*/*v*)	HaCaT cells	In vitro enhancement of antimicrobial peptide’s mRNA expression.	0.02% hesperidin; 70% ethanol (*w*/*v*)	Cosmetics/ Cosmeceuticals	Increase in epidermal differentiation-related protein expression (loricrin and filaggrin); β-glucocerebrosidase and glutathione reductase activity regulation	[84,85]
.	1.03 mmol	In vitro non-cellular assays	Radical scavenging activity.	Hesperidin-conjugated pectins: pectin aqueous solution of 8 g (2.5 wt%); hesperidin intermediate (1.03 mmol) solution into a 7.5 wt% sodium hydroxide solution and epichlorohydrin (1.03 mmol) Hesperidin-conjugated pectins Hydrogels: pectin conjugates (5 wt% pectin solution) individually crosslinked with Ca^2+^, Zn^2+^, and Fe^3+^ (0.22 mM ionic solutions)	Cosmetics/ Cosmeceuticals	Hesperidin’s ion-binding ability induced the crosslinking of the hydrogel conjugates	[86]
Commercial hesperidin	0.05% (*w*/*w*)	In vitro non-cellular assays	Formulation’s bioadhesive properties improvement.	Biocomposites of cellulose, collagen, and hesperidin (4:1:0.05 (*w*/*w*))	Cosmetics/ Cosmeceuticals	N/A	[87]

HDFs—Human Dermal Fibroblasts; FTS—Full Thickness Skin models; HaCaT—Adult Human Keratinocyte; PM2.5—Particulate Matter with an aerodynamic diameter less than 2.5 µm, studied as an air-pollutant testing model; N/A—Non-Added.

### 4.2. UVA and UVB Radiation-Induced Skin Damage

Sun damage, caused by regular sun exposure, is the main catalyst of premature skin aging, inducing a condition known as “skin’s photoaging” [75,88]. This phenomenon is mediated by UV radiation, which can be divided into ultraviolet A (UVA), ultraviolet B (UVB), and ultraviolet C (UVC) rays. The main components of UV radiation that reach and negatively affect skin are the UVA and UVB radiations. The characteristic wavelength range of each of these components is 320–400 nm (UVA) and 280–320 nm (UVB). For instance, skin disorders such as inflammation and erythema can be caused by chronic exposure to UV rays [75]. Additionally, photoaging is related to abnormal epidermal thickness and overall connective tissue disorganization [78,79]. On a cellular level, solar radiation might induce apoptosis and generate intra- and/or extracellular ROS, both leading to DNA damage [75,78]. In particular, excessive ROS production led by UVB can disrupt cellular equilibrium by increasing oxidative stress and causing several negative alterations, such as protein carbonylation, besides the aforementioned alterations [75].

In the same way that hesperidin from orange peels is characterized as a compound with multifaceted activities for targeting skin aging, it is also a promising molecule to counteract sun damage within this tissue [89]. Therefore, among other modes of action (Table 2), it is claimed that hesperidin can restrain UV effects by reducing epidermal thickening, by regulating the expression of angiogenesis-related factors (e.g., PI3K/Akt, VEGF, HIF-1α, and MMPs), by modulating apoptotic proteins induced by oxidative stress, and by regulating some immune cells and inflammatory cytokines, contributing to decreased inflammation [75,88,89,90]. Furthermore, Lacatusu and coworkers [91] stated that topical hydrogels formulated with nano-lipid carriers (NLCs), loaded with hesperidin, showed a remarkable photoprotective effect by absorbing 99% of UVB and 83% of UVA radiation. Hence, hesperidin might be a suitable sunscreen ingredient, for instance, when incorporated into cosmetic or cosmeceutical formulations aiming to target UVA and UVB radiation-induced skin damage (Table 2) [88].

### 4.3. Skin’s Hyperpigmentation and Depigmentation Conditions

Skin conditions that imply the alteration of the natural individual’s skin color can be suggestive of a hyperpigmentary disorder, such as melasma or solar lentigo, or even depigmentary autoimmune diseases, such as vitiligo [66,82,94]. Moreover, since these disorders are highly perceptible, they can represent a source of low self-esteem and discomfort to the patients.

Melanin is the molecule that defines the color of hair and skin in humans while holding an important protective effect against UV light and ROS [66,82]. In general, skin hyperpigmentation-related disorders occur upon an increase in melanin synthesis, also known as melanogenesis, or in its delivery, which happens in the melanosomes. Melanosomes are the organelles responsible for melanogenesis and for the packing of melanin in melanocytes, which are then transferred to keratinocytes [66,72]. Melanogenesis, itself, is regulated by numerous inner molecular (e.g., tyrosinase) and environmental (e.g., UV rays) factors. Tyrosinase and microphthalmia-associated transcription factor (MITF), for instance, hold major roles in melanogenesis [82]. Therefore, the downregulation of these molecules has been suggested as a target for melanin regulation in hyperpigmentary conditions [66,82]. On the contrary, the suppression of factors such as the melanocyte-stimulating hormone (MSH) or oxidative stress can lead to depigmentation, causing depigmentary skin conditions [94]. Overall, melanogenesis has been studied to treat hyperpigmentation- and depigmentation-related diseases, leading to the development of products to target these issues [82].

The understanding and modulating of the skin pigmentation process have been attracting interest for a long time regarding skincare industrial applications [72]. Thus, it is crucial to find new molecules that can work toward these objectives by regulating melanogenesis and that can be successfully incorporated into novel skincare formulations.

Hesperidin from orange peels, as an antioxidant molecule, has shown both anti-melanogenic and melanogenic activities (Table 3), depending on its application, by interacting with different melanogenic intermediates [45,82,94]. In the case of hesperidin, it was demonstrated that it can inhibit melanosome transport by blocking the Rab27A–melanophilin, which is a protein complex that takes part in this pathway, on mammalian epidermal melanocytes [72,82]. Hesperidin can also downregulate the MITF protein, by activation of the MEK/ErK1/2 pathway, leading to low tyrosinase levels, which ends up in the reduction in melanin production [82]. On the other hand, flavonoids can also work to induce topical re-pigmentation when formulated properly. This can be particularly useful in the case of diseases such as vitiligo by hesperidin’s immunomodulation. Despite the mechanism not being well understood, Shivasaraun and colleagues [94] suggested that the combination of flavonoids, such as hesperidin, with trimethylpsoralen, in a nanoemulsion-based gel formulation, can improve the treatment of vitiligo.

Therefore, hesperidin might be a useful ingredient to be applied either in skin lighting or re-pigmentation products for cosmetic and cosmeceutical purposes.

### 4.4. Wound Healing

Chronic wounds such as ulcers and burns are conditions that require long periods of time to heal (usually over three months) or that may never heal completely due to the interruption of the natural wound-healing process, which is kept at the inflammatory stage. These kinds of wounds are often caused by the patient’s inefficient immunity system, which is usually linked to some type of disease (e.g., diabetes), infection, or metabolic deficiency, affecting the individual’s quality of life [95,96,97]. In this type of wound, the excessive production of edema exudate is common. Exudate overproduction can lead to damage to healthy skin around the wound and can potentiate delayed healing by keeping the wound in the inflammation stage for long periods of time [96,97]. This happens due to an abnormal expression of growth factors, TIMPs, and MMPs, as well as impaired integrins’ regulation, and ROS formation. These alterations end up affecting the activity and differentiation of fibroblasts and keratinocytes, preventing the formation of a stable ECM [98]. Furthermore, these types of wounds can lead to more serious issues like hemorrhages, severe infections, or even gangrene. In this way, chronic wounds deserve further attention, since they represent a major healthcare problem around the world [99].

Wound healing is a complex and dynamic process that is necessary for the recovery and renewal of skin after any type of damage (chemical or physical) [98]. Overall, this process can be divided into three stages, namely: (i) inflammation and hemostasis; (ii) proliferation with ECM deposition; and (iii) re-epithelization with tissue remodeling [96]. Since it is an extremely intricate process, many types of factors, enzymes, and cells are involved in wound healing [97].

In addition to the above-mentioned biological features, flavonoids like hesperidin extracted from orange peels also exhibit anti-inflammatory and antibacterial properties (Table 4), indicating that it can act as a therapeutic molecule, and it holds the potential to be incorporated into skincare pharmaceuticals [80,95,100]. Regarding wound healing, some studies have been carried out to understand hesperidin’s mechanisms of action. Some of the proposed mechanisms are related to free radicals scavenging, pro-inflammatory cytokines (e.g., IL-1β, IL-8, and TNF-α) suppression, and enhancement of cell division in fibroblasts [95].

Formulation-wise, Bagher and colleagues [95] described that in an in vivo injured skin model, hesperidin-loaded alginate/chitosan hydrogel helped to enhance and accelerate wound healing. Concerning hesperidin’s bioactivities, those results were achieved by epithelialization stimulation, the deposition of ECM components, and cellular proliferation [95]. Sujitha et al. [101] also recognized that hesperidin nanoemulgel holds ideal characteristics to be applied as a topical drug delivery system. Moreover, hesperidin in wounds from diabetes-induced rats showed promising effects, which might be due to angiogenesis modulation via VEGF-c, Smad-2/3 mRNA, and Ang-1/Tie-2 expression [102].

Hence, hesperidin formulations and scaffolds present potential to be applied in the wound healing and skincare therapeutics fields.

### 4.5. Skin Cancer and Other Cutaneous Diseases

Skin cancer is one of the most prevalent cancers in the human population, particularly in fair-skinned individuals. The frequency of cutaneous cancer has been increasing, and they can be divided into different types according to their primary site on the skin (e.g., melanoma, basal cell carcinoma) [73,113,114]. Research related to the development of drugs to treat skin cancer is focused on specific factors and cellular mechanisms, such as DNA damage targeting, apoptosis, and restraining the proliferation of cancerous cells, as well as reducing the increased angiogenesis, which is typical of the carcinogenesis process [73,89,115]. Consequently, new innovative treatments based on natural compounds have been explored [73].

Kouassi et al. [12] described that polyphenols can act against skin cancer. Flavonoids, such as hesperidin from citrus fruits (Table 5), are among the natural molecules that present antitumoral activity [75,91]. Moreover, it is believed that this flavone might target skin carcinogenesis through ROS scavenging by provoking alterations in the mitochondrial membrane’s potential and eventually causing cell necrosis in cancerous cells [72,73,116]. For instance, Zhao and colleagues [73] studied the effect of hesperidin in A431 cells (a skin cancer cell line), where they observed that after exposure to the flavone, the cell cycle is arrested in the S phase, which is proposed to be mediated by its downregulation of cyclin D, CD1K2, and thymidylate synthase. Moreover, they reported the change in Apf-1 caspase-3, caspase-8, and PARP protein expression after hesperidin treatment. Furthermore, Li et al. [115] stated that hesperidin reduced the increased tumor cell proliferation by disturbing aerobic respiration in a time- and dose-dependent manner.

Apart from skin cancer, other relevant skin diseases affect the human population. Conditions such as rosacea, psoriasis, and atopic dermatitis, among others, are highly impactful in patients’ daily life [89,117,118].

In this way, hesperidin has also been claimed to have a positive impact in helping ameliorate those conditions as a therapeutic agent (Table 5). For example, rosacea and psoriasis are characterized by an increased angiogenesis rate that, as mentioned before, can be inhibited by hesperidin through the regulation of factors such as the PI3K/Akt signaling pathway, VEGF and HIF-1α [89,117]. Furthermore, Gupta et al. [119] and Nagashio et al. [118] demonstrated some beneficial therapeutic features for the skin with orally administered hesperidin. Moreover, these studies demonstrated that the oral intake of hesperidin helped to treat pigmented purpuric dermatosis lesions in a human case report [119] and to treat atopic dermatitis in an animal testing model [118]. Additionally, hesperidin was suggested to regulate hair follicle growth in androgenetic alopecia, which is achieved through the phosphorylation of Src, Akt, and AMP kinase, and by NO release in the skin. These molecular interactions result in vasculature regrowth, leading to the growth of the hair follicle [120].

Thus, hesperidin seems to hold major potential for skin cancer and disease treatment and might be useful for skincare pharmaceutical therapies incorporation, leading to promising results when combined with pre-established or new treatments, in order to maximize its bioactivities potential.

**Table 5:** Biological activities of commercial and orange peel’s hesperidin related to skin cancer and other skin diseases.

Hesperidin/ Extract Type	Hesperidin Levels Tested	Testing Model	Main Biological Properties	Formulation	Potential Applications	Other Relevant Properties/ Molecular Pathways	Ref.
Sweet orange peel extract	0.05–1 mg/mL	*A. flavus* *A. parasiticus* *A. niger* *A. ochraceous* *F. proliferitum* *P. verrucosum* BJ-1 cells Cancerous cell lines (HCT-116/MCF7/HepG2)	Liposome protection against UV-induced peroxidation; DNA protection activity; Free hesperidin presents pro-oxidant activity against cancerous cells; Antifungal activity; Growth inhibition of MCF-7 and HepG-2; NPs reduced cytotoxic effects against normal cells (BJ-1).	Nanoparticles (NPs): hesperidin (16.5 g) and PEG (50 g)	Skincare Pharmaceutical/ Therapeutic Agent	Hesperidin-loaded NPs enhanced protection capacity against DNA damage (maybe due to the alteration of the free hesperidin’s delivery, inside the cells, at the site of action)	[64]
Commercial hesperidin	10–50 μM	Epidermoid carcinoma cell line (A431 cells)	Decreasing in A431 viability and colony-forming potential in a dose-dependent manner; A high number of A431 cells in the S phase; Augmented ROS levels, increased cytosolic Ca^2+^ level, and reduced mitochondrial membrane potential level in A431; DNA breakage and non-apoptotic cell death inducing in A431.	N/A	Skincare Pharmaceutical/ Therapeutic Agent	Cyclin D, CDK2, and thymidylate synthase expression decreased in A431 cells; Reduced ATP levels by up to 40% in A431 cells	[73]
Commercial hesperidin	In vivo (oral intake): 125–500 mg/Kg/day In vitro: 5–20 μg/mL	Mouse HaCaT cells	Reduced keratinocyte excessive proliferation and ameliorated abnormal differentiation of epidermal cells in in vitro psoriasis-like-induced model; Reduced the pathological changes of psoriasiform dermatitis by reducing localized inflammatory cytokine expression in vivo; Reduce excessive cell proliferation and differentiation in skin lesions in a dose-dependent manner in vivo.	N/A	Skincare Pharmaceutical/ Therapeutic Agent	Inhibition of tumor cell proliferation in a time/dosage-dependent manner; Hesperidin was found to be present in the *stratum corneum* and upper *spinous* layer after each dose	[115]
Commercial hesperidin	Formulation’s oral intake: 100–400 mg/Kg/day	Swiss albino mice	Reducing of the tumor incidence in a dose-dependent manner; Reduced average number of tumors in a dose-dependent manner; Reduced neoplastic transformation in skin cells.	Hesperidin in saline water containing 0.5% (*w*/*v*) carboxymethyl-cellulose	Skincare Pharmaceutical/ Therapeutic Agent	Catalase and SOD activity enhancement in skin tumors after 24 weeks of treatment; GSH levels and activity increase in a dose-dependent manner; Reduction in the expression of Rassf7, Nrf2, PARP, and NF-κB genes in a dose-dependent manner; Inhibition of MDA in skin tumors, in a dose-dependent manner, leads to a lipid peroxidation decrease	[116]
Commercial hesperidin	Oral intake: 0.1% (*w*/*w*)	Splenocytes from NC/Nga mice	No atopic dermatitis-induced skin lesions observed after treatment; Clinical scores (evaluation of atopic dermatitis symptoms) of hesperidin-fed mice increased until 10 weeks of age.	N/A	Skincare Pharmaceutical/ Therapeutic Agent	Increase in IgE serum levels; Decrease in IFN-c, IL-17 and IL-10 levels	[118]
Hesperidin from micronized purified flavonoid fraction	Oral intake: 50 mg	Human 45-year-old male (case report)	Treatment improved the patient pigmented purpuric dermatoses lesions, after 2 weeks.	Oral intake: diosmin (450 mg), hesperidin (50 mg), *E. prostata* extract (100 mg), and calcium dobesilate (500 mg)	Skincare Pharmaceutical/ Therapeutic Agent	Surface expression of monocyte or neutrophil CD62L reduction; Leukocyte activation inhibition; VEGF expression downregulation; Decreasing of TNF-α, MMPs and NGAL expression; Production of oxygen free radical and lipid peroxidation inhibition	[119]
Hesperidin from orange waste	5–45 μmol/L	A375 cells CHL01 cells SKMEL147 cells	DPPH scavenging activity; No cytotoxicity to all cell lines at the tested concentrations.	Nanostructured lipid carriers: cupuaçu butter mixed with buriti oil, anhydrous lanolin (10% *v*/*v*), and L-hesperidin (4 mmol)	Skincare Pharmaceutical/ Therapeutic Agent	N/A	[121]

HCT-116—colorectal adenocarcinoma cell line. MCF-7—mammary adenocarcinoma cell line. HepG2—hepatocellular carcinoma cell line. A431—epidermoid carcinoma cell line. A375/CHL01/SKMEL147—human melanoma cell lines; N/A—non-added.

## 5. Concluding Remarks

This review critically examined research conducted on hesperidin extracted from orange peels, a by-product of agro-food waste, between the years 2012 and 2022. Within this topic, the present work aimed to focus on the extraction methods of hesperidin, as well as on its bioactivities, with a view to potential industrial applications in skincare. The studies on the orange peels originating from the waste of the agro-food industry brought attention to the benefits of giving a “new life” to these by-products as valuable resources for obtaining value-added products, aligning with the principles of the circular economy. In this way, this review aims to provide an overview of the research evolution within this topic and to uncover the innovations made in terms of hesperidin’s bioactivities application on the skin and the formulations developed in the last decade.

Between the years of 2012 and 2022, 87 relevant articles were analyzed in depth, with 32 related to the extraction and purification methods of hesperidin from orange peels, 46 focusing on hesperidin’s potential bioactivities on the skin, and the remaining being reviews regarding the latter subjects. Moreover, by analyzing the issues related to “Biological Activities on Skin”, it is possible to understand specifically the areas where hesperidin has attracted more interest concerning possible skincare applications. Furthermore, an increasing trend can be observed in the number of articles published throughout the years related to this matter. In total, 256 articles were obtained upon the applied search criteria; however, many of them focused either on another type of source besides orange peels or were related to other polyphenols or applications. Therefore, further studies are needed with a focal point on the bioactivities of hesperidin from orange agro-food wastes, particularly orange peels, leading to skin applications.

Another aspect to be mentioned is that despite research efforts to clarify the bioactivities of hesperidin’s molecular mechanisms of action, envisaging potential applications, this question remains only partly explored. These assessments are particularly important since this molecule presents remarkable characteristics to be utilized in numerous industrial applications, from food to cosmetics and healthcare fields. Furthermore, hesperidin seems to have the capacity to be eventually incorporated into more complex formulations or even combined with other compounds from agro-food wastes in a way to potentiate its activity.

This review outlines the evolution and current trends of the extraction methods and bioactivities of hesperidin from orange peels obtained during the processing of oranges. Further research regarding this topic can be highly valuable, since it might imply the development of new impactful skincare formulations incorporating hesperidin that can help with the treatment of multiple skin disorders. While exploring and potentiating hesperidin bioactivities, studies under the scope of the present review can implement a reduced waste ethic by the valorization of peels from oranges.

## Figures and Tables

**Table 2:** Biological activities of commercial and orange peel’s hesperidin, related to UVA and UVB radiation-induced skin damage.

Hesperidin/ Extract Type	Hesperidin Levels Tested	Testing Model	Main Biological Properties	Formulation	Potential Applications	Molecular Pathways/ Other Relevant Properties	Ref.
Commercial hesperidin	0.0125–1 mM	HaCaT cells	ROS scavenging activity increases in a dose-dependent manner; UVB absorption activity; DNA protection against UVB; Optimal hesperidin concentration: 50 μM.	N/A	Cosmetics/ Cosmeceuticals	Mitochondrial membrane depolarization regulation by apoptotic pathways inhibition; Caspase-3, caspase-9, and BAX downregulation; Upregulated expression of Bcl-2; Prevention of protein oxidation against UVB-induced ROS	[75]
Commercial hesperidin	10–320 μg/mL	HaCaT cells	Cell viability increases in a concentration-dependent manner; Cell growth enhancement after inhibition by UVA; Enhancement of SOD activity; Reduction in ROS.	N/A	Cosmetics/ Cosmeceuticals	Reduction in MDA content; Downregulation of TNF-α mRNA/ protein, IL-1β mRNA/protein, and IL-6 mRNA/protein expression; Increase in T-AOC levels; Potential as a sunscreen agent	[88]
Commercial hesperidin	5–20 μM	HEKs cells HDFs cells	In vitro inhibition of UVB-induced angiogenesis.	N/A	Cosmetics/ Cosmeceuticals	Repression of MMP-9, MMP-13, and VEGF expression; Regulation of MEK/ERK and PI3K/Akt pathways	[89]
Hesperidin from *Citrus unshiu*	20 µg/mL	HaCaT cells	Regulation of inflammatory response.	N/A	Cosmetics/ Cosmeceuticals	Downregulation of IL-8 and TNF-α mRNA/protein expression; Inhibition of NF-ƙB/IƙBα signal cascade; Inhibition of p38 MAPK phosphorylation; Inhibition of COX-2 activation	[90]
Commercial hesperidin	0.3% (*w*/*v*)	In vitro non-cellular assays	Inhibition of short-life radicals; Inhibition of ABTS radical; Absorption of 99% UVB radiation and 83% UVA radiation.	NLCs: amaranth oil; pumpkin seed oil; 7% UVA filter; diethylamino hydroxybenzoyl hexyl benzoate; 7% UVB filter; ethylhexyl salicylate; and 3% hesperidin. NLCs-Carbol gel (ratio 1:1) cosmetic formulation: final composition of 3.17% diethylamino-hydroxybenzoyl hexyl benzoate, 3.17% ethylhexyl salicylate; and 1.13% hesperidin	Cosmetics/ Cosmeceuticals	In vitro, hesperidin presented a burst pattern with an accelerated release from the NLCs	[91]
*Citrus sinesis* L. peel extract	500–1000 mg of extract	In vitro non-cellular assays	In vitro inhibition activities of DPPH radical, elastase and collagenase.	NLCs optimal formulation: cocoa butter/olive oil and 1000 mg extract Extract-NLCs cream: clove (*Eugenia caryophyllus*), nagarmotha (*Cyperus scariosus*), tulsi (*Ocimum sanctum*), nutmeg (*Myristica fragrans*), linseed (*Linum usitatissium*), wheat grains, cereals (*Triticum aestivum*), neem (*Azadirachta indica*), ethanol, stearic acid, potassium hydroxide, sodium carbonate, glycerin, water, perfume	Cosmetics/ Cosmeceuticals	N/A	[3,92]
Commercial hesperidin	N/A	In vivo trials: dermatological assessment, instrumental skin analysis and satisfaction survey	High antioxidant activity; Absorption of UVA, HEV, and IR radiation; Skin barrier functions for protection; Reduction in erythema, skin irritability and visibility of telangiectasia.	Cosmetic product containing hesperidin and SPF 50+	Cosmetics/ Cosmeceuticals	N/A	[93]

HaCaT—Adult Human Keratinocyte; HDFs—Human Dermal Fibroblasts; HEKs—Human Epidermal Keratinocytes; NLCs—Nanostructured Lipid Carriers; N/A—Non-Added.

**Table 3:** Biological activities of commercial and orange peel’s hesperidin, related to skin’s hyperpigmentation and depigmentation conditions.

Hesperidin/ Extract Type	Hesperidin Levels Tested	Testing Model	Main Biological Properties	Formulation	Potential Applications	Molecular Pathways/ Other Relevant Properties	Ref.
*Citrus mitis blanco* peel water extract	Fraction reach in hesperidin 0.5 mg/mL	In vitro non-cellular assays	Tyrosinase inhibitory activity (IC50 was 3.3 mg/mL).	N/A	Cosmetics/ Cosmeceuticals	N/A	[41]
Citrus peel extract	1–50 µg/mL	Melan-a cells	Antioxidant activity DPPH in a dose-dependent manner; Inhibition of tyrosinase activity; Decrease in melanin content in Melan-a in a dose-dependent manner.	N/A	Cosmetics/ Cosmeceuticals	Extract antioxidant activity was greater than vitamin C (control), at the same dosage; Melanogenesis inhibition through suppression of melanosome transport in melanocytes	[45]
*C. unshiu* peel-press cakes ethanolic extract	86 mg/g	B16F10 cells HaCaT cells	No cytotoxic effect on both cell types; Decrease in cellular melanin content and tyrosinase activity in a dose-dependent manner.	N/A	Cosmetics/ Cosmeceuticals	Reduction in α-MSH-stimulated tyrosinase, MTIF protein, TRP-1 and TRP-2 expression in a dose-dependent manner	[66]
Commercial hesperidin	0.1–500 μM	B16F10 cells Neonatal Human Melanocytes	No cytotoxic effect up to 40 μM, on both cell types; Reduction in melanin content and tyrosinase activity, in a dose-dependent manner; Radical scavenging activity against DPPH.	N/A	Cosmetics/ Cosmeceuticals	Decrease in tyrosinase, TRP-1 and TRP-2 proteins in a dose-dependent manner; Suppression of melanogenesis through MTIF downregulation and activation of Erk pathways	[81]

B16F10—Murine Melanoma Cell Line; Melan-a—Mouse Melanocyte Cell Line; HaCaT—Human Keratinocyte Cell Line; N/A—Non-Added.

**Table 4:** Biological activities of commercial and orange peel’s hesperidin related to wound healing potential.

Hesperidin/ Extract Type	Hesperidin Levels Tested	Testing Model	Main Biological Properties	Formulation	Potential Applications	Bioavailability/Molecular Pathways/Other Relevant Properties	Ref.
Commercial hesperidin	1–10% (*w*/*v*)	3T3 cells Wistar rats *S. aureus* *P. aeruginosa*	Hemocompatible; Antimicrobial; Cell proliferation and collagen synthesis increase in a dose-dependent manner; In vivo enhanced performance of would therapy in a dose-dependent manner; Granulation tissue and epidermal proliferation; Wound contraction, epidermal layer formation and remodeling	Alginate/Chitosan/ Hesperidin Hydrogel: 2:1 (*v*/*v*) alginate and chitosan solutions (sodium alginate (2% (*w*/*v*)) in deionized water; chitosan (2% (*w*/*v*)) in 0.5% (*v*/*v*) acetic acid) + hesperidin (1 or 10% weight of polymer Alg/Chit) + calcium chloride 50 mM (CaCl_2_) and 10 μL glutaraldehyde with NaOH 1 M (crosslink).	Skincare Pharmaceutical/ Therapeutic Agent	Neovascularization enhancement in a dose-dependent manner; between 8.9 and 17.2% of hesperidin has been released within the first 3 to 6 h, followed by a sustained release of 77.03 ± 8.71%, over 14 days	[95]
Commercial hesperidin	10 mg/mL	Ex vivo goat skin Wistar albino rats	Improvement of therapeutical treatment for anti-inflammatory activity; The gels were close to a neutral pH (6.8), presenting a low risk of skin irritation	Optimal emulsion formulation: 100 mg stearic acid; 50 mg cholesterol, 125 mg soya lecithin; 100 mg hesperidin in 10 mL ethanol. Optimal topical nanoemulgel: Ratio 1:1 of Carbopol to hydroxypropylmethyl cellulose.	Skincare Pharmaceutical/ Therapeutic Agent	Potential as a carrier for topical drug delivery system; the hesperidin release from the optimal emulsion was 98.6% after 6 h; regarding ex vivo permeation studies of hesperidin nanoemulgel, the cumulative drug that permeated through the skin was 98.9% after 4 h	[101]
Commercial hesperidin	25–100 µg/mL ^1^	Ex vivo rat skin	Hemocompatible; Acceleration of wound closure in a dose-dependent manner; Reduction in inflammation and infection; Improvement of wound contraction, epidermal layer formation, remodeling, and collagen synthesis, in a dose-dependent manner	Hesperidin-loaded PAMAM Dendrimer (Hsp-PAMAM): hesperidin at 2.5, 5, 7.5, or 10% (*w*/*v*) was loaded into PAMAM dendrimer. Hsp-PAMAM based hydrogel bandages: sodium alginate; deionized water; chitosan solution and acetic acid.	Skincare Pharmaceutical/ Therapeutic Agent	Safe and compatible for topical delivery; hesperidin shows an outburst pattern in the first 5 h, followed by delayed release, from the bandages; after 24 h, 86.367% of hesperidin was released; rat skin showed a deposition of the drug in the epidermis up to 15–25 µm; the drug was conserved in between the epidermis and dermis, which is ideal for full-thickness wound therapy	[102]
Commercial hesperidin	5% (*w*/*v*)	Swiss albino mice	Wound-healing acceleration; Enhancement of wound contraction; Induction of cell proliferation	Hydrogel: hesperidin (5 g); deionized water (10 mL) and polyethylene glycol 400 (PEG) (380–420 g/mol).	Skincare Pharmaceutical/ Therapeutic Agent	Increased nitric oxide, glutathione and SOD levels; repression of NF-kB and COX-2	[103]
Commercial hesperidin	0.5% (*w*/*w*)	Dermal fibroblasts from donated human skin	Fibroblasts proliferation induction; Migration without terminal differentiation and collagen synthesis; Increased progression of wound confluence and closure	Niacinamide (3.0% *w*/*w*), L-carnosine (1.0% *w*/*w*), hesperidin (0.5% *w*/*w*) and Biofactor HSP^®^ (0.05% *w*/*w*).	Skincare Pharmaceutical/ Therapeutic Agent	N/A	[104]
Commercial hesperidin	30–120 mM	*S. aureus* *E. coli* HUVECs cells Sprague–Dawley rats	Antibacterial; DPPH scavenging activity; No significant cytotoxicity; Cell proliferation and migration activity improvement; Acceleration of wound closure after infection by *S. aureus*; Re-epithelization enhancement; Stimulation of collagen synthesis and deposition; Stimulation of angiogenesis and hair follicle synthesis	Nanoparticles: silver nitrate (AgNO_3_) (2 mL, 3.397 mg/mL) and hesperidin solution (10 mL, 17.6 mg/mL). Note: The nanoparticles were Incorporated into a hydrogel.	Skincare Pharmaceutical/ Therapeutic Agent	Activation of basic fibroblast growth factor (bFGF) and Stirt 1 expression; suppression of the expression of pro-inflammatory factors (NF-ƙB, MMP9, TNF-α, and IL-6)	[105]
Commercial hesperidin	Formulation’s oral intake: 50 mg/kg/day	Sprague–Dawley rats	Necrosis reduction in epidermis and dermis; No congestion or hemorrhage, after 14 days	Oral intake: Bacitracin combined with hesperidin.	Skincare Pharmaceutical/ Therapeutic Agent	Decrease in IL-1 beta and TNF-α levels	[106]
Commercial hesperidin	0.5% (*w*/*w*)	Sprague–Dawley rats	Reduction in wound surface area; Increased wound contraction; Potentiation of wound epithelization by the 28th day; Promotion of cellular infiltration and proliferation	Scaffold: collagen in 0.05-M acetic acid (0.6% (*w*/*w*)), chondroitin-6-sulfate (in 0.05-macetic acid). Scaffolds in cosmeceutical formulation: niacinamide (3.0% *w*/*w*), L-carnosine (1.0% *w*/*w*), hesperidin (0.5% *w*/*w*) and Biofactor HSP^®^(0.05% *w*/*w*). Scaffolds crosslinking: 1-ethyl-3-(3-dimethyl aminopropyl)-carbodiimide and N-hydroxysuccinimide (EDAC/NHS) and 0.5% glutaraldehyde (GA).	Skincare Pharmaceutical/ Therapeutic Agent	N/A	[107]
Commercial hesperidin	Oral intake: 25–100 mg/kg/day	Sprague–Dawley rats	Wound half-closure time improvement in a dose-dependent manner; Oxido-nitrosative stress reduction; Hydroxyproline levels (collagen synthesis marker) increase; Angiogenesis and re-epithelization induction	N/A	Skincare Pharmaceutical/ Therapeutic Agent	VEGF-c, Ang-1, Tie-2, TGF-β and Smad 2/3 mRNA expression upregulation	[108,109]
Commercial hesperidin	Oral intake: 10–80 mg/kg/day	Rats	Wound healing promoted in Diabetes-induced animals; Wound half-closure time improvement	N/A	Skincare Pharmaceutical/ Therapeutic Agent	Reduction in MDA, MPO, TNF-α, and IL-6 levels in a dose-dependent manner; stimulation of VEGF, GSH, HDP, and SOD expression in a dose-dependent manner	[110]
Commercial hesperidin	5–10% (*w*/*w*)	Swiss albino mice	Epithelization time reduction; Enhancement of wound contraction; Wound-healing activity improved in a dose-dependent manner in the *S.aureus* infected wound model (antibacterial activity)	Ointments containing: 5% (*w*/*w*) or 10% (*w*/*w*) hesperidin.	Skincare Pharmaceutical/ Therapeutic Agent	N/A	[111]
Commercial hesperidin	50–250 µg/mL ^2^	In vitro non-cellular assays	Strong DPPH scavenging activity	Nanoparticles optimal formulation: hesperidin (15 mg); chitosan (20 mg); soya lecithin (10 mg) and surfactant (1 mL)	Skincare Pharmaceutical/ Therapeutic Agent	In vitro, hesperidin presents an outburst pattern in the first 4 h followed by delayed release from nanoparticles; the optimal formulation was stable, safe to use and could improve the topical bioavailability of hesperidin due to its nano-size with a larger surface area; the formulation is a suitable hesperidin delivery agent, leading to improved wound healing	[112]

^1^ Concentrations of hesperidin-loaded PAMAM dendrimer-based hydrogel bandages; ^2^ Concentrations of hesperidin-loaded nanoparticles. 3T3—Murine fibroblast cell line. HUVECs—Human umbilical vein endothelial cells; N/A—Non-added.

## Data Availability

The data presented in this study are available on request from the corresponding author.
